# Phenotypic and fitness consequences of plasticity in the rhythmic replication of malaria parasites

**DOI:** 10.1098/rstb.2023.0340

**Published:** 2025-01-23

**Authors:** Jacob G. Holland, Aidan J. O'Donnell, Alejandra Herbert-Mainero, Sarah E. Reece

**Affiliations:** ^1^ Institute of Ecology and Evolution, School of Biological Sciences, University of Edinburgh, Edinburgh EH9 3FL, UK; ^2^ Eccles Institute of Human Genetics, University of Utah, Salt Lake City, UT 84112, USA; ^3^ Institute of Immunology and Infection Research, School of Biological Sciences, University of Edinburgh, Edinburgh EH9 3FL, UK

**Keywords:** intraerythrocytic developmental cycle, *Plasmodium*, periodicity, T-cycle, replication rhythm, fitness

## Abstract

The environments that parasites experience within hosts change dramatically over 24 h. How rhythms shape host–parasite–vector interactions is poorly understood owing to the challenges of disentangling the roles of rhythms of multiple interacting species in the context of the complex lifecycles of parasites. Using canonical circadian clock-disrupted hosts, we probe the limits of flexibility in the rhythmic replication of malaria (*Plasmodium*) parasites and quantify the consequences for fitness proxies of both parasite and host. We reveal that parasites alter the duration of their replication rhythm to resonate with host rhythms that have short (21 h) daily T-cycles as accurately as when infecting hosts with 24 h cycles, but appear less capable of extending their replication rhythm in hosts with long (27 h) cycles. Despite matching the period of short T-cycle hosts, parasites are unable to lock to the correct phase, likely leading to lower within-host productivity and a reduction in transmission potential. However, parasites in long T-cycle hosts do not experience substantial fitness costs. Furthermore, T-cycle duration does not affect disease severity in clock-disrupted hosts. Understanding the rhythmic replication of malaria parasites offers the opportunity to interfere with parasite timing to improve health and reduce transmission.

This article is part of the Theo Murphy meeting issue issue ‘Circadian rhythms in infection and immunity’.

## 1. Introduction

Daily rhythms pervade interactions between organisms and their environments, affecting biological processes, spanning from gene expression to physiology, behaviour and population dynamics. To capitalize on opportunities offered by daily environmental rhythms and to cope with the constraints they impose, organisms have evolved to schedule their activities adaptively, usually via circadian clocks. Patterns of rhythmicity can sometimes be constrained by phylogeny, and yet many organisms display remarkable potential to plastically adjust rhythms in response to environmental changes [1,2]. For example, nocturnal rodents are usually active in the dark phase (night) to minimize exposure to daytime predators but become diurnal when nights are too cold to pay the energetic costs of foraging [3], or when nighttime competition for food is high [4]. These observations can be explained by integrating the concepts of chronobiology with evolutionary theory for adaptive phenotypic plasticity and for life histories to explain variation in daily rhythms. We follow this approach to interrogate rhythmicity in host–parasite interactions, focusing on malaria infections. Within their hosts, parasites (including pathogens, microbes, etc.) are exposed to myriad daily environmental rhythms that regulate cyclic opportunities such as the availability of nutritional resources, and dangers such as rhythms in immune defences [5]. Rhythms in the biotic environment are vulnerable to perturbation, occurring, for example, as a result of a mismatch between the phenology of predators or prey [6]. Hosts experience disrupted rhythms during infections [7], suggesting that ‘embodied organisms’ such as parasites may require flexibility in their rhythms to cope with variation in the rhythms they experience during infections.

*Plasmodium* parasites—the causative agents of malaria—are famously rhythmic, undertaking synchronous cycles of replication in the host’s blood that causes fever when they simultaneously burst every 24, 48 or 72 h, depending on the species [8]. During each cycle of asexual replication, termed the intraerythrocytic development cycle (IDC), parasites progress through morphologically and functionally distinct stages (commonly referred to as the ring, trophozoite and schizont stages) within the vertebrate host’s red blood cells. Schizonts complete each cycle by synchronously releasing (merozoite) progeny to begin a new IDC. The rodent model *P. chabaudi* aligns its IDC with the timing of the host’s feeding–fasting rhythms [9–12]. This schedule is thought to allow the parasite to capitalize on the appearance of nutrients in the blood following the host’s rhythmic foraging [11]. Furthermore, aligning to host rhythms appears to ensure that short-lived transmission forms are most infective during the night, when *Anopheline* mosquito vectors typically forage for blood [13–15]. Despite the fitness benefits of replicating in-step with the 24 h circadian rhythms of hosts and vectors, the timing (phase), duration (period) and synchrony (amplitude) of the IDC rhythm vary across *Plasmodium* species [8,16] and during infections [17] in manners that are not explained by variation in the rhythms of hosts. Explaining variation in fitness-related traits is a major aim of evolutionary biology, and there are several reasons why the IDC rhythm might vary. A key step in understanding the evolution of the IDC rhythm involves asking how variation in the IDC rhythm impacts parasite and host fitness. For example, different degrees of synchrony within the IDC of the human malaria parasite *P. falciparum* may be explained by a theory predicting that low synchrony is favoured when there is a risk of highly genetically related parasites inadvertently interfering with each other’s ability to simultaneously secure resources [18,19]. This scenario predicts that genetically homogenous infections and/or scarcity of resources select for parasites to be less synchronous *per se*, or to plastically reduce synchrony when these conditions are encountered.

Recognizing that host–parasite interactions are dynamic over multiple time scales brings ecological realism to explanations for parasite phenotypes and can uncover hitherto unknown parasite strategies that interventions can exploit [20]. Here, we explore plasticity in the IDC rhythm and its consequences for parasite and host fitness. Specifically, we exploit flexibility in rhythms of canonical circadian clock-disrupted hosts that allow experimental perturbation of the duration (period; ‘T-cycles’) of host rhythms to examine parasite responses. We show that clock-disrupted hosts deficient for period 1 and period 2 (*Per1/2-*null [21,22]) generally mask to T-cycles with 21, 24 and 27 h periodicities (to which wild-type mice are unable to entrain), and we track parasite performance during infections as well as characterizing the IDC schedule and infection severity. We predicted that because perturbations to the timing of the IDC rhythm relative to the phase of host rhythms cause *P. chabaudi* to reschedule by accelerating the IDC [10], it is more likely to achieve alignment with hosts in a short T-cycle and these parasites will suffer fewer fitness consequences than when faced with hosts in a long T-cycle. Determining the fitness consequences of rhythms for parasites and hosts is timely and important given that plasticity in the IDC schedule helps *Plasmodium* tolerate antimalarial drugs [20,23] and that the temporal selective landscape of malaria parasites is changing because malaria-vectoring mosquitoes are evading bed nets by altering the time of day when they forage for blood [24–26].

## 2. Methodology

Our first experiment established a mouse model for generating host T-cycles shorter than, equal to or longer than 24 h within the same host genotype. Our second experiment characterized parasite IDC rhythms in hosts subject to different T-cycles and followed infections throughout their duration to quantify parasite fitness proxies and infection severity.

### (a) Experiment 1: mouse strains and manipulating T-cycles

The canonical TTFL (transcription–translation feedback loop) clock is present in cells throughout the body and is normally entrained by external rhythmic factors such as light, allowing biological functions to be synchronized to environmental rhythms [27,28]. We predicted that compared to wild-type mice, mice with a disrupted TTFL clock are more likely to align to non-24 h T cycles, since they are not restricted by entrainment mechanisms of the TTFL that act to coordinate rhythms with a period of approximately 24 h (even in the absence of external rhythmic stimuli) across organs. TTFL clock-disrupted mice are able to align at least some biological functions (including locomotor activity, rest and foraging) with external rhythmic factors such as light (termed ‘masking’; [29]), when experiencing 24 h rhythmic light–dark cycles [30].

To assess the entrainment limits of wild-type mice and the masking ability of mice with a disrupted TTFL clock, we compared the locomotor activity rhythms of male, 8–10 week-old wild-type and age-matched TTLF-disrupted *Per1/2*-null mice (see electronic supplementary material, Mouse strains and conditions). Following acclimation to light : dark 12 h : 12 h (LD 12 : 12) (lights on 07.00–19.00 UTC+1), we assigned mice to a TcShort treatment (*Per1/2-*null *n* = 3, wild type *n* = 5) or a TcLong treatment (*Per1/2*-null *n* = 4, wild type *n* = 5). We fitted all mice with radio-frequency identification (RFID) tagged probes, and then each cage of mice was placed on an RFID reader baseplate to monitor locomotor activity and body temperature (see electronic supplementary material, Mouse tracking system). After beginning activity monitoring, mice remained in LD 12 : 12 (lights on 07.00–19.00 UTC+1) for six cycles (TcShort mice) or seven cycles (TcLong mice). The subsequent dark phase was either shortened to 10.5 h (TcShort) or lengthened to 13.5 h (TcLong) to begin the new Tc photoschedules, and thereafter mice were kept at LD 10.5 : 10.5 (TcShort = 21 h) or LD 13.5 : 13.5 (TcLong = 27 h), respectively. We continued to monitor locomotor activity for 15 (TcShort) or 11.5 cycles (TcLong).

### (b) Experiment 2: design and infection

Having established that, unlike wild-type mice, all *Per1/2*-null mice achieve the correct period and exhibit nocturnality in both short and long photoschedules (see §3a; electronic supplementary material, figures S1,S2), we used this strain as hosts for the main experiment. Male and female mice (14–20 weeks old) were randomly allocated to each of three T-cycle (Tc) treatment groups: Tc21, Tc24 (lights on 14.30–02.30) and Tc27. All mice were given 11 days to acclimate to their T-cycle treatments, and to ensure all received the same parasite inoculum, all hosts were infected when the time of lights on coincided across all T-cycles.

We verified there were no differences in baseline measures of weight and red blood cell (RBC) density between treatment groups on the day before infection (weight, *F*_2,40_ = 0.04, *p* = 0.96; RBC, *F*_2,40_ = 2.28, *p* = 0.12). All hosts were infected with 1 × 10^5^ *P. chabaudi* (genotype DK, Malaria Reagents Repository http://www.malariaresearch.eu/ at the University of Edinburgh) parasitized red blood cells via intravenous injection. Within each T-cycle treatment, we established two cohorts of infections: one to characterize host rhythms over a short time series (‘tagged’ cohort, females) and the second to characterize the IDC rhythm and quantify parasite fitness proxies and infection severity (‘sampled’ cohort, males). Using different mice for these data types allowed host rhythms to be tracked via subcutaneous RFID passive integrated transponder (PIT) tags without being interrupted by the handling of animals necessary to sample infections [31]. Previous studies suggest *P. chabaudi* exhibits equivalent infection dynamics in male and female *Per1/2*-null hosts and allocating one sex to each cohort enabled animals to be housed in the most ethical manner (group housing) while avoiding stressful disruption to social dynamics [32]. The sampled cohort consisted of *n* = 10 mice for Tcs 21 and 27 and *n* = 9 for Tc24, and the tagged cohort contained *n* = 5 mice for Tcs 21 and 27 and *n* = 4 for Tc24. Because parasite rhythms are the focus of the study, we allocated more mice to the sampling cohort to prioritize statistical power for comparing IDC rhythms and parasite/host fitness proxies. We also simultaneously infected a third cohort consisting of wild-type mice (WT24; C57BL/6 J males; *n* = 6), housed in the same conditions as the Tc24 sampling mice, to test whether host genotype, under a normal (24 h) T-cycle duration, affects the IDC rhythm, parasite performance and disease severity.

### (c) Data collection

#### (i) Tagged cohort

We collected locomotor activity and body temperature data spanning the same 335 h of continuous tracking for all mice in each Tc treatment, which began at 244 h before infection (34.5 h after the beginning of experimental photoschedules) and ended at 91 h post infection (HPI). Tags in two mice in the Tc27 treatment failed and so these individuals did not contribute locomotor activity or temperature data. We examined video recordings (made by the Actual HCA system, Actual Analytics) for each cage over 2.5 cycles immediately prior to infection (Tc21 = 50.5 to 8.0 h; Tc24 = 50.5 to 0.5 h; Tc27 = 68.0 to 0.5 h, before infection, respectively) to investigate feeding rhythms. Specifically, we recorded the number of mice feeding (defined as head pressed to food in the food tray) at each whole minute, binned into 60 min intervals, to calculate the mean number of feeding bouts per hour. Note that because feeding rhythms are characterized for each cage, rather than for each individual, it is not possible to generate standard errors when visualizing the rhythm.

#### (ii) Sampled cohort

**Figure 1.**
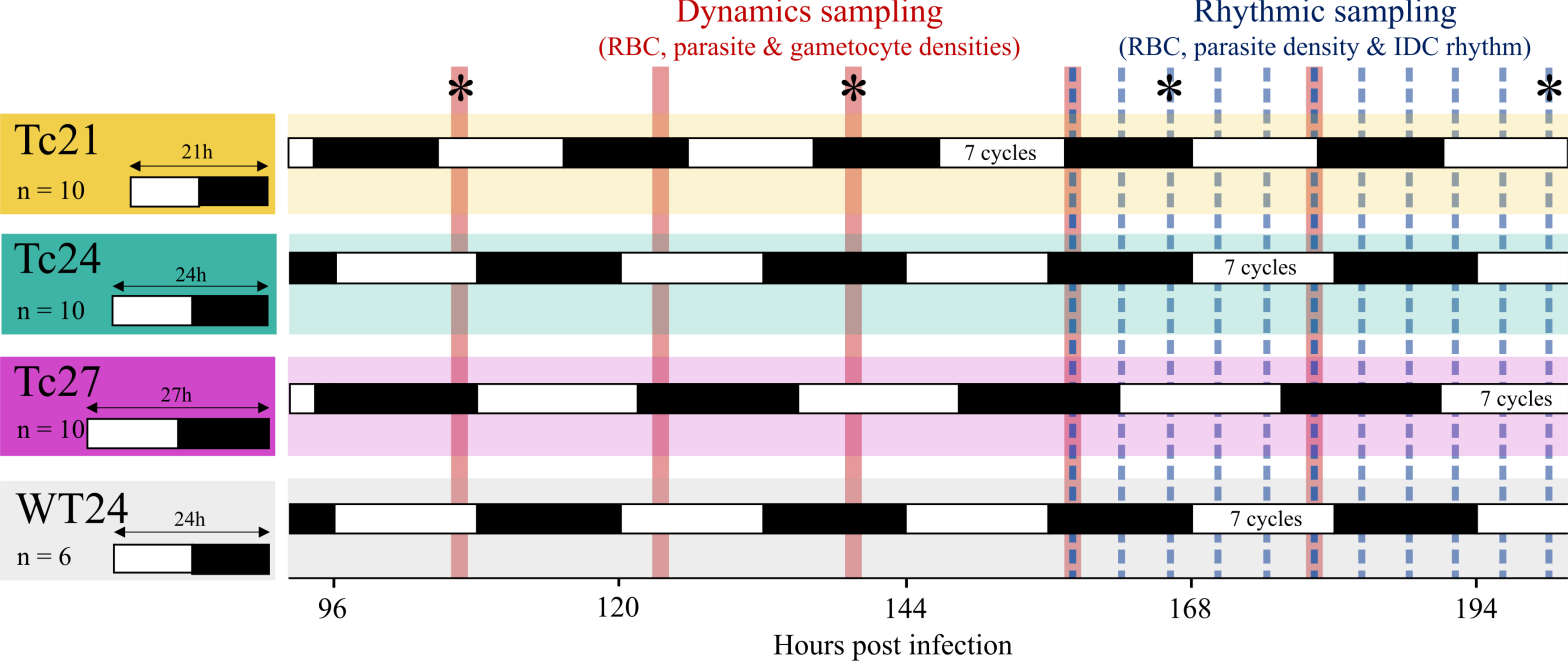
Experimental design. Clock-disrupted mice (*Per1/2-*null) in three different photoschedules corresponding to day lengths (T-cycles) of 21 h (Tc21, LD 10.5 : 10.5), 24 h (Tc24, 12 : 12) and 27 h (Tc27, 13.5 : 13.5), as well as wild-type mice (WT24, LD 12 : 12), were infected with *Plasmodium chabaudi* when the beginnings of their light phases coincided. Sampling cohorts had infection dynamics monitored by quantifying host RBC count and parasite and gametocyte densities at five ‘dynamics sampling’ timepoints (red lines), scheduled to allow fair comparison between treatments for both equivalent HPI and the number of LD cycles. During the final two LD cycles, the same mice were also subjected to ‘rhythmic sampling’ (blue dashed lines) every 4 h over 40 h to characterize IDC rhythms. Host weight was measured at timepoints indicated by asterisks. White/black bars indicate light/dark periods for each treatment, with hours post infection shown on the bottom axis (time before, or during the first 90 h of infection is not shown).

We designed our sampling regime to allow comparison of parasite performance (densities of asexually replicating IDC stages and of sexual transmission forms), disease severity (impact on weight and RBC density) and characteristics of the IDC schedule, at points representing the same HPI and/or equivalent number of LD cycles (figure 1). For example, timepoint 1 corresponded to 106.5 HPI for all treatments, whereas timepoints 1, 2 and 3 corresponded to approximately 5 LD cycles (5.07–5.17) for the Tc21, Tc24 and Tc27 treatments, respectively. We sampled blood from the tail vein and processed it to determine IDC stage distribution, RBC densities, total parasite densities and gametocyte densities [33,34], depending on the variables measured at each sampling point (figure 1; see electronic supplementary material, Sampling and data collection). We completed sampling at 198.5 HPI, just prior to peak parasitaemia, to prevent the onset of severe symptoms, host rhythm disruption and acquired immunity from confounding the direct impact of host T-cycles on parasites. All procedures were carried out in accordance with the UK Home Office regulations (Animals Scientific Procedures Act 1986;SI 2012/ 3039) and approved by the University of Edinburgh.

### (d) Analyses

We used R for all statistical analyses [35]. To characterize the rhythmicity of host locomotor activity and host body temperature, we used Lomb Scargle (hereafter ‘LS’) periodograms for each mouse to identify significantly (*α* = 0.05) rhythmic hosts, setting minimum and maximum periods as 20–28 h. We then re-ran and integrated LS periodograms for rhythmic hosts using the Fisher method to give single parameter values of period and amplitude (defined as MESOR to peak) for each treatment (‘group-level data’, using the ‘meta2d’ or ‘meta3d’ functions in the ‘MetaCycle’ R package, [36]). We next modelled the effects of T-cycle treatment on period length or amplitude using the ‘lmer’ function in the ‘lme4’ R package [37]. To compare locomotor activity and body temperature between the light and dark phases, for each treatment, we modelled the effect of light versus dark on locomotor activity and on body temperature. To characterize feeding rhythms for each treatment, we used an LS periodogram for each cage (i.e. T-cycle treatment), as well as verification by the ‘JTK_CYCLE’ method (via MetaCycle [36]), which was possible since the data were binned into whole hours. We compared feeding between light and dark phases within each treatment using Wilcoxon rank sum tests. To characterize the rhythmicity of parasite IDCs, we first identified significantly (*α* = 0.05) rhythmic infections using harmonic regression analysis (Clocklab, Actometrics). We then ran and integrated LS periodograms for rhythmic infections, which acted as verification for rhythmicity and allowed us to use the resultant values to model the effects of T-cycle treatment on period and amplitude, as above. To determine the phase of the proportion of ring stages, we re-ran LS periodograms, fixing the period equal to T-cycle length (which allowed the phase to be interpretable relative to light and dark), and used the resultant values to model the effect of T-cycle treatment on phase, as above.

To compare host health variables and parasite fitness proxies across T-cycle treatments, we constructed models using the ‘lmer’ R function [37]. For parasite dynamics models (total parasite density or gametocyte density), we excluded all zeros from the data and log-transformed it, and used fixed predictor terms for treatment, HPI and LD cycle number, as well as interactions between treatment and each of the other two variables in the model set. For parasite cumulative density models (total parasites or gametocytes), we summed the density over all timepoints for each mouse, including zeros in the data, and used the natural log of this value as the response variable and a fixed predictor term for treatment. For the host disease severity models (weight and RBC density), we similarly fitted models with fixed predictor terms for treatment and LD cycle number, as well as timepoint and timepoint × treatment interaction (i.e. a factor rather than continuous predictor for HPI, given the nonlinear dynamics of the data). Finally, for maximum weight/RBC loss (initial value minus lowest value), we used a fixed predictor term for treatment. For all models where more than one datapoint was collected per mouse, we included a random intercept term for mouse ID to account for repeated measures. We checked that all models conformed to assumptions using the ‘DHARMa’ R package [38] and respecified the model if necessary until assumptions were met. To determine the most parsimonious model in each model set (including the null model) based on small-sample corrected Akaike Information Criterion (AICc), and model weights, we used the ‘dredge’ function in the ‘MuMIn’ R package [39]. Within the most parsimonious model for each model set, we used *t*-tests fitted by Satterthwaite’s method to determine the significance of each predictor. For model specifications, see electronic supplementary material, tables S1–S7.

## 3. Results

### (a) Experiment 1: *Per1/2*-null, but not wild type mice align to non-24 h T-cycles

#### (i) Locomotor activity

All mice exhibited locomotor rhythms in LD 12 : 12, with peak activity during the dark phase (electronic supplementary material, figure S1). Specifically, locomotor activity was significantly rhythmic (LS periodograms, *α* = 0.05) for all *Per1/2*-null mice in TcShort and TcLong photoschedules (*p* < 0.001) with period estimates close to 24 h (*Per1/2*-null TcShort = 24.74 h, *Per1/2*-null TcLong = 24.19 h; electronic supplementary material, figure S2a,c). Locomotor activity was also rhythmic in all WT mice (*p* < 0.001) with period estimates close to 24 h (WT TcShort = 24.04 h, TcLong = 24.29 h; electronic supplementary material, figure S2b,d).

Following the shift to TcShort (21 h) and TcLong (27 h) T-cycles for 11–15 cycles, respectively, all *Per1/2*-null mice exhibited locomotor activity rhythms (3/3 TcShort and 4/4 TcLong, *p* < 0.001) with period estimates matching their new T-cycle durations (TcShort = 21.03 h, TcLong = 27.08 h, electronic supplementary material, figure S2a,c) and their activity peaked during the dark phase (electronic supplementary material, figure S1a,b). All wild-type mice also exhibited locomotor activity rhythms (5/5 TcShort, 4/4 TcLong, *p*<0.001), but their period estimates (TcShort = 23.82 h, TcLong = 26.01 h, electronic supplementary material, figure S2b,d) did not match T-cycle duration as closely as those of *Per1/2*-null mice. Furthermore, while *Per1/2*-null mice were mostly active at night, peak activity either drifted between each cycle (TcShort, electronic supplementary material, figure S1c) or switched to the light phase (TcLong, electronic supplementary material, figure S1d) for wild-type mice. The different responses of *Per1/2*-null and WT mice resulted in an interaction between mouse strain and T-cycle treatment as the best explanation for period estimates of locomotor activity rhythms (ΔAICc of the model with treatment and strain only = 19.4, model weight = 0.01).

#### (ii) Body temperature

As for locomotor activity, all mice exhibited rhythms in body temperature in LD 12 : 12 (electronic supplementary material, figure S3, period estimates; *Per1/2*-null TcShort = 24.12 h, *Per1/2*-null TcLong = 24.08 h, WT TcShort = 24.27 h, WT TcLong = 24.02 h) and were warmer during the dark phase (electronic supplementary material, figure S4). Following the shift to TcShort (21 h) and TcLong (27 h) T-cycles for 11–15 cycles, respectively, all mice remained rhythmic (*p* < 0.001). Period estimates for *Per1/2*-null mice were close to their T-cycle durations (TcShort = 21.03 h, TcLong = 27.08 h, electronic supplementary material, figure S3a,c) but the WT mice (TcShort = 23.57 h, TcLong = 26.24 h, electronic supplementary material, figure S3b,d) did not match T-cycle duration. Unlike wild-type mice, *Per1/2*-null mice in all T-cycles exhibited higher body temperature in the dark phase. The different responses of *Per1/2*-null and WT mice resulted in an interaction between mouse strain and T-cycle treatment as the best explanation for period estimates of body temperature rhythms (ΔAICc of the model with treatment and strain only = 5.82, model weight = 0.05).

### (b) Experiment 2: host rhythms in non-24 h T-cycles

**Figure 2.**
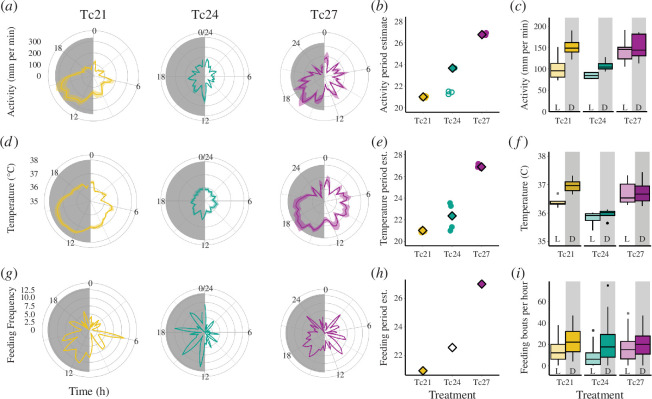
Characterization of locomotor activity (*a,b,c*), body temperature (*d,e,f*) and feeding (*g,h,i*) rhythms of *Per1/2*-null hosts across T-cycle (Tc) treatments: 21, 24 and 27 h. Circular coordinate plots (*a,d,g*) depict the levels of each metric around the clock, where 360° corresponds to a complete cycle of light (white hemisphere) and dark (grey hemisphere) over 21, 24 or 27 and with the same (wall clock) times labelled on the *x* (circular) axis of each plot: 0, 6, 12, 18 and 24 (where 0 represents the beginning of the light phase). Means (solid lines) and s.e.m. (ribbons) over successive days are plotted, with the distance from the centre representing the level of each metric (*y* axes). Point plots (*b,e,h*) depict the periods (wavelength) of rhythms for each T-cycle treatment estimated from Lomb Scargle periodograms, with circles for individual mice and diamonds for group-level values (filled shapes indicate significant rhythms and open shapes represent non-significant rhythms). Box and whisker plots (*c,f,i*) depict the median and quartiles for each metric during the light (L) versus the dark (D) for each T-cycle treatment. Feeding was measured for whole cages, precluding individual values for estimating errors (*g,h*). Individual host periodograms for locomotor activity and body temperature are presented in electronic supplementary material, figure S5.

Our first set of analyses established that host rhythms in non-24h T-cycles were consistent with those observed in our first experiment. Locomotor activity was significantly rhythmic (LS periodograms, *α* = 0.05) in 5/5 Tc21, 1/4 Tc24 and 3/3 Tc27 mice (electronic supplementary material, figure S5a). Significant rhythmicity was also detected in the group-level analysis (Tc21, *p* < 1 × 10^−6^; Tc24, *p* < 0.001; Tc, *p* < 1 × 10^−6^, figure 2*a*), with period estimates very similar to T-cycle durations: 21.01 h for Tc21, 23.66 h for Tc24 and 26.75 h for Tc27 (figure 2*b*). For significantly rhythmic mice, the period was also best explained by T-cycle treatment (ΔAICc of null model = 50.4, model weight = 1.00; electronic supplementary material, table S2; figure 2*b*). Amplitude was not affected by T-cycle treatment, since the null model (without treatment predictor term) was best supported (ΔAIC of the model with treatment = 6.89, model weight = 0.03; electronic supplementary material, table S2). For each T-cycle treatment, the bulk of locomotor activity occurred at night (nocturnal), with a model including whether lights were on/off being more parsimonious than the null model in each case (Tc 21, ΔAICc of null model = 196.7, model weight = 1.00; Tc24, ΔAICc of null model = 35.7, model weight = 1.00; Tc27, ΔAICc of null model = 3.98, model weight = 0.88; figure 2*a*,*c*, electronic supplementary material, table S2).

Body temperature was significantly rhythmic (LS periodograms, *α* = 0.05) in 5/5 Tc21 mice, 4/4 Tc24 mice and 3/3 Tc27 mice (electronic supplementary material, figure S5b). As we found for locomotor activity, rhythms were also detected at the group level (Tc21 *p* < 1 × 10^−6^; Tc24, *p* < 1 × 10^−6^; Tc27, *p* < 1 × 10^−6^; figure 2*d*), with period estimates similar to T-cycle durations: 21.01 h for Tc21, 22.36 h for Tc24 and 26.92 h for Tc27 (figure 2*e*). For significantly rhythmic mice, the period was best explained by T-cycle treatment (ΔAIC of null model = 23.2, model weight = 1), again with periods similar to T-cycle durations (electronic supplementary material, table S2). Amplitude was also marginally best explained by T-cycle treatment (ΔAIC of null model = 2.51, model weight = 0.78), with amplitude for Tc21 mice being more than twice that of Tc24 mice, while amplitude for the Tc27 mice was also higher than for the Tc24 mice (electronic supplementary material, table S2). This indicates that temperature is more variable than locomotor activity in non-24h T-cycles. For the Tc21 and Tc24 treatments, the mean temperature was higher at night, with a model including lights on/off status being more parsimonious than the null model (Tc21, ΔAICc of null model = 392, model weight = 1; Tc24, ΔAICc of null model = 27.7, model weight = 1; electronic supplementary material, table S2; figure 2*d*,*f*). However, for the Tc27 treatment, the mean temperature was almost equivalent between light and dark and the null model was the most parsimonious (ΔAICc of the model with light status = 6.38, model weight = 0.96). In this group, body temperature peaks early in the dark phase and is at its nadir at the end of the dark (figure 2*d*; electronic supplementary material, table S2).

Feeding patterns were also rhythmic for mice in the Tc21 treatment (LS periodogram, *p *< 0.002; JTK_CYCLE, *p* = 0.003; figure 2*g*) with a period of 20.95 h (LS) or 21.0 h (JTK_CYCLE). Tc24 mice did not have a significantly rhythmic feeding pattern (LS periodogram, *p* = 0.99, JTK_CYCLE, *p* = 0.99) and nor did mice in the Tc27 treatment according to the LS periodogram (*p* = 0.17) but they had a marginally rhythmic feeding pattern according to the JTK_CYCLE (*p* = 0.0498), with a period of 27.0 h (figure 2*h*). Nonetheless, most importantly, feeding in all T-cycle treatments tended to peak during the first half of the dark phase, with smaller peaks during the light phase, and with significantly more feeding during the dark than the light (figure 2*i*) in the case of the Tc21 and Tc24 treatments (Wilcoxon’s rank sum tests; Tc21, *W* = 521, *p* = 0.006; Tc24, *W* = 643, *p* = 0.005; Tc27, *W* = 687, *p* = 0.150).

Overall, the experimental hosts followed nocturnal rhythms (see §2a), providing parasites with ecologically relevant condensed or elongated versions of 24 h environmental rhythms. Unexpectedly, most of the Tc24, and a few of the Tc27 hosts, exhibited weaker locomotor activity and body temperature rhythms than reported previously and than we observed in experiment 1. It is possible that being infected exacerbated the negative effects of a loss of circadian regulation (which may include metabolic inefficiencies), resulting in *Per1/2*-null mice being unable to pay the energetic costs of maintaining high levels of activity throughout an elongated dark phase and also struggling to fast for an elongated light phase, dampening their rhythms. Nonetheless, mice in all T-cycle treatments were nocturnal, and we note that basing our statistical analysis on light versus dark differences (figure 2*c*,*f*,*i*) is a conservative approach to characterizing the nocturnal patterns evident in the polar plots (figure 2*a*,*d*,*g*). More importantly, all hosts masked sufficiently to undertake the bulk of their foraging at night (figure 2*g*,*i*); the phase of feeding–fasting provides the strongest known time-cue for the IDC schedule [9–12] and the IDC rhythm followed the same qualitative patterns in Tc24 and WT24 hosts (see §3c). Taken together, these results suggest that Tc24 hosts provided an adequate control group for other T-cycles within the same host genotype.

### (c) Experiment 2: parasite IDC rhythms resonate with only 24 and 21 h T-cycles

**Figure 3.**
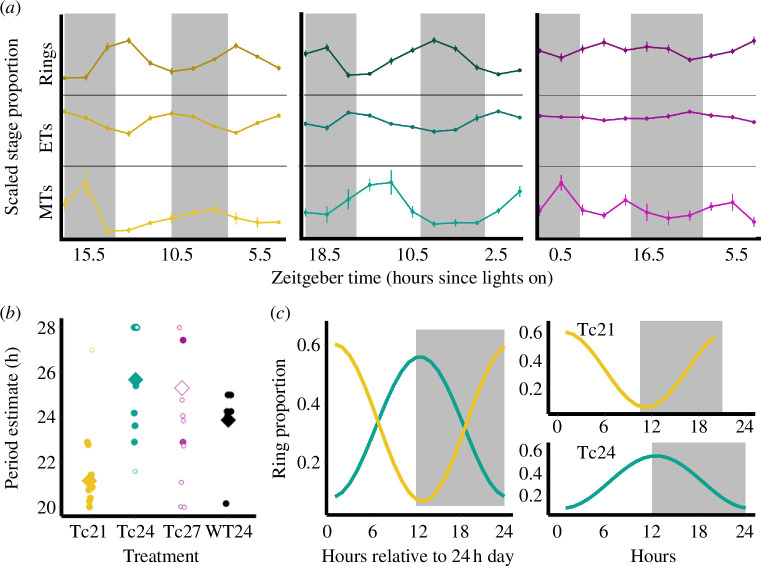
The intra-erythrocytic developmental cycle (IDC) rhythm of *Plasmodium chabaudi* in *Per1/2*-null hosts across Tc treatments: 21, 24 and 27 h. Ridge plots (*a*) depict the proportions of the three most common IDC stages (rings, ET = early trophozoites, MT = mid trophozoites) for each treatment over 36 h. Each IDC stage is scaled to the same height to aid the visibility of their respective rhythms. Points/lines, means; error bars, s.e.m. Ring stage rhythms for each infection are presented in electronic supplementary material, figure S6. The point plot (*b*) depicts the periods detected by Lomb Scargle periodograms, with circles for individual mice and diamonds for group-level values (filled shapes indicate significant rhythms and open shapes represent non-significant rhythms), and WT24 infections are included for comparison to Tc24. Waveform plots (*c*) depict the ring stage rhythm in Tc21 (yellow) and Tc24 (green) treatments (the IDC in Tc27 was not rhythmic) generated by Lomb Scargle periodograms. In the left panel, the Tc21 wave is scaled to 24 h to allow phase comparison (where white/grey bars indicate light/dark) and the right panels illustrate a single cycle (with the period fixed to 21 or 24 h) for each treatment. Note that peaking at the end of the light phase in the Tc24 group was unexpected because rings normally peak at the end of the dark in this host–parasite combination [9–12]. White/grey bars indicate periods of light/dark, respectively.

Our second set of analyses quantified and compared the parasites’ IDC rhythmicity across T-cycle treatments, and also compared Tc24 infections with those in WT24 hosts (electronic supplementary material, figure S6). The IDC was rhythmic (harmonic regression, *α* = 0.05) in 9/10 Tc21, 7/9 Tc24, 2/9 Tc27 and 6/6 WT24 infections. Analysing significantly rhythmic infections at the group level verified significant rhythms in three treatments (LS periodograms; Tc21, *p* = 0.002, *q* = 0.011; Tc24, *p* = 0.009, *q* = 0.044; WT24, *p* = 0.003, *q* = 0.013), with periods of 21.17 h for Tc21, 25.67 h for Tc24 and 23.87 h for WT24 (figure 3*a*). Supporting the findings from individual infections, the IDC of parasites in Tc27 hosts was not significantly rhythmic in the group analysis (Tc27, *p* = 0.147, *q* = 0.684), and so, all Tc27 infections are considered arrhythmic hereafter (figure 3*a*). The most parsimonious model for the periods of significantly rhythmic infections showed that the period was best explained by treatment (ΔAICc of null model = 93.3, model weight = 0.99; figure 3*b*; electronic supplementary material, table S3).

The group analyses using significantly rhythmic infections also revealed similar amplitudes across treatment groups: 0.19 for Tc21, 0.17 for Tc24 and 0.21 for WT24. The model including T-cycle treatment was not better than the null model (ΔAICc = 2.45, null model weight = 0.77; electronic supplementary material, table S3). For comparison, the group LS periodogram analysis of all IDCs in the (arrhythmic) Tc27 group estimated a much lower ‘amplitude’ value of 0.08. All treatments had similar proportions of stages averaged across timepoints (rings 29.1–33.2%, early trophozoites 58.8–65.0%, mid trophozoites 5.3-6.8%, late trophozoites 0.6–1.9%, schizonts < 0.1%), suggesting that the dampening of the IDC in the Tc27 group is owing to asynchronous replication, not by the IDC stalling at a particular developmental stage.

To compare the phase between the three rhythmic treatments, we set periods equal to T-cycle durations (21 or 24 h) since the phase (relative to the light cycle) is not comparable when the period differs from the T-cycle length. The peak phase was 1.85/21 (=2.11/24) for Tc21 and 14.45/24 for Tc24 infections, which is equivalent to an 11.66 h difference in a 24 h cycle (i.e. almost complete antiphase; figure 3*c*). For comparison, the phase of the IDC for WT24 infections was very similar to Tc24, at 14.03/24. The most parsimonious model showed that phase was best explained by T-cycle treatment (ΔAICc of null model = 26.3, model weight > 0.99), corresponding again to nearly a 12 h difference between Tc21 and Tc24 infections (coefficient ± s.e.m. = −12.38 ± 0.64; electronic supplementary material, table S3).

### (d) Experiment 2: T-cycle duration affects parasite fitness proxies

Our third set of analyses compared proxies for within-host survival (total density of parasites) and transmission potential (density of gametocytes) across T-cycle treatments. Our comparisons allowed us to assess whether parasite performance was affected by the rhythmicity of the IDC as well as host genotype, by comparing Tc24 *Per1/2-*null and WT24 wild types. We compared temporal dynamics in two ways: according to the absolute age of infections (HPI) or the number of LD cycles experienced, as well as the cumulative densities of parasites, across T-cycle treatments.

**Figure 4.**
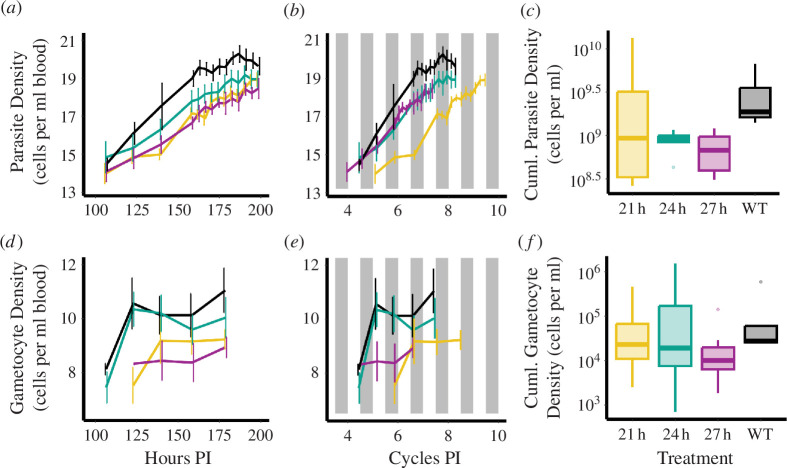
Total densities of *Plasmodium chabaudi* parasites (*a,b,c*) and gametocytes (i.e. transmission stages; (*d,e,f*) in *Per1/2*-null hosts across Tc treatments: 21 (yellow), 24 (teal) and 27 (purple) hours, and wild-type hosts (black). Line plots (*a*) and (*d*) depict densities for each treatment as a function of the absolute age of infections (HPI), whereas (*b*) and (*e*) depict density as a function of the number of LD cycles post infection. White/grey bars depict light/dark and means ± s.e.m. are plotted. Box plots in (*c*) and (*f*) depict cumulative parasites and gametocytes, with medians (horizontal lines), interquartile ranges (boxes), range (whiskers) and outliers (points) plotted. Note that timepoints differ for total parasites and gametocytes owing to the sampling regime (see figure 1).

Parasite density dynamics werebetter explained by the number of LD cycles than by HPI and also varied by T-cycle treatment, but the trajectories had similar slopes across Tc treatments (i.e. no interactions with LD cycle or HPI; model weight = 0.969; figure 4*a*,*b*; electronic supplementary material, table S4). Specifically, for all treatments, density increased by 277% each LD cycle (coefficient of log values ± s.e. = 1.33 ± 0.03; *t* = 40, d.f. = 388, *p* < 1 × 10^−15^). When comparing each group with the reference Tc24 group, the intercept for the Tc21 group was significantly (86%) lower (coefficient of log values = −1.99 ± 0.52; *t* = −3.8, ḍ.f. = 31, *p* < 0.001), but the Tc27 (9% higher; coefficient of log values = 0.08 ± 0.52; *t* = 0.2, d.f. = 31, *p* = 0.87) and WT24 (105% higher; coefficient of log values = 1.21 ± 0.60; *t* = 2, d.f. = 31, *p* = 0.052) groups did not differ. When comparing the cumulative number of parasites across T-cycles, the model including T-cycle treatment was not better than the null model (ΔAICc = 4.81, null model weight = 0.92; figure 4*c*). This suggests that density is lower for Tc21 parasites at a given LD cycle, but that the greater number of LD cycles experienced by these parasites throughout the infection monitoring window means they are able to reach a similar density overall.

Similarly, gametocyte dynamics was better explained by the number of LD cycles than by HPI, and also varied between T-cycle treatments, but the trajectories had similar slopes (i.e. no interactions between treatment and LD cycle or HPI; model weight = 0.805; figure 4*d*,*e*; electronic supplementary material, table S5). Specifically, for all treatments, gametocyte density increased by 102% each LD cycle (coefficient of log values ± s.e. = 0.70 ± 0.13, *t* = 5.3, d.f. = 69, *p *< 1 × 10^-6^; figure 4*d*). When comparing each group to the reference Tc24 group, the intercept for the Tc21 group was significantly (89%) lower (coefficient of log values = −1.55 ± 0.69; *t* = −2.3, d.f. = 31, *p* = 0.027), but the Tc27 (56% lower; coefficient of log values = −0.81 ± 0.68; *t* = −1.1, d.f. = 27, *p* = 0.25) and WT24 (81% higher; coefficient of log values 0.60 ± 0.70; *t* = 0.9, d.f. = 23, *p* = 0.40) groups did not differ significantly. However, including T-cycle treatment in the model for cumulative gametocyte density was not better than the null model (ΔAICc = 4.67, null model weight = 0.91; figure 4*f*). As we found for total parasite density, this suggests that gametocyte density is lower for Tc21 (and possibly Tc27) parasites at a given LD cycle, but the greater number of LD cycles experienced by these parasites throughout their infections means they reach a similar density overall.

### (e) Experiment 2: T-cycle duration does not affect disease severity

**Figure 5.**
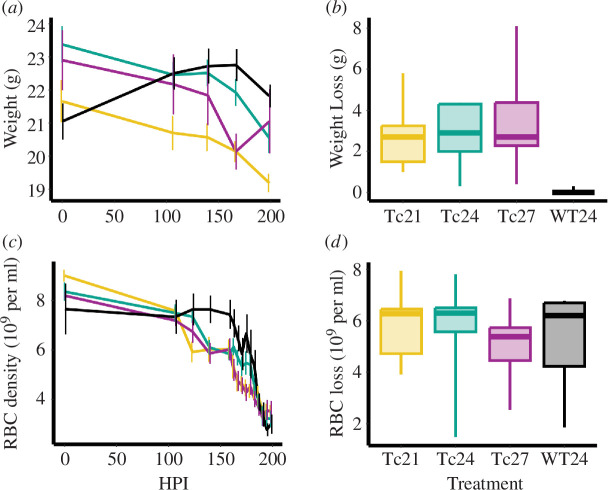
Disease severity of *Plasmodium chabaudi* parasites in Per1/2-null hosts across Tc treatments: 21 (yellow), 24 (teal) and 27 (purple) hours, and wild-type hosts (black). Host weight (grams) is shown as a function of the hours post infection (*a*), and maximum weight loss since the beginning of infection (*b*). Host red blood cell (RBC) density (10^9^ per ml of blood) is shown as a function of the hours post infection (*c*), and maximum RBC density loss since the beginning of infection (*d*). Line plots in (*a*) and (*c*) depict means ± s.e.m. Box plots in (*b*) and (*d*) depict medians (horizontal lines), interquartile ranges (boxes) and ranges (whiskers).

Our fourth set of analyses tested whether T-cycle treatment affected the severity of symptoms experienced by hosts. The most parsimonious model to explain host weight dynamics included timepoint, T-cycle treatment and their interaction, but not the number of LD cycles (model weight = 0.54; electronic supplementary material, table S6), suggesting that HPI better explained host weight dynamics. The interaction between timepoint and T-cycle treatment was mostly driven by the WT24 group; weight declined during infections for all the *Per1/2*-null hosts (figure 5*a*), but WT24 hosts exhibited an initial increase in weight (likely because their younger age meant they were growing during the experiment), and weight began to recover by the end of the experiment in the Tc27 group. Accounting for differences in absolute weight, by comparing maximum weight loss, revealed that the most parsimonious model included a treatment predictor variable (model weight = 0.97), although again this effect was mostly driven by the WT24 group in which the maximum weight loss was much lower (coefficient ± s.e., relative to Tc24, Tc21 = −0.25 ± 0.75, Tc27 = 0.36 ± 0.75, WT24 = −2.85 ± 0.86; figure 5*b*).

Analogous to weight dynamics, the most parsimonious model to explain RBC density dynamics included timepoint, T-cycle treatment and their interaction, as well as the number of LD cycles (model weight = 0.52, electronic supplementary material, table S7), suggesting that HPI was more relevant. Specifically, RBC loss was experienced by all groups over time, albeit later in the WT24 treatment (likely owing to the health benefits of their initial weight gain), and some of this decline was attributed to the number of LD cycles experienced (−0.48 × 10^9^ RBC per ml for each LD cycle). However, subtle variation between treatments in RBC dynamics did not translate to an overall impact on RBC maximum loss, since the most parsimonious model to explain maximum RBC density loss did not include a treatment term (null model weight = 0.96, ΔAICc = 6.34; figure 5*d*).

## 4. Discussion

We demonstrate that clock-disrupted *Per1/2*-null mice are generally capable of ‘masking’ to a ‘standard’ 24 h (LD 12 : 12) T-cycle and unlike wild-type mice they are also capable of matching substantially shorter (21 h) and longer T-cycle (27 h) durations (figure 2; electronic supplementary material, S1−S5). We also reveal sufficient plasticity in the IDC to align with a short (21 h) T-cycle, but in hosts with a longer T-cycle (27 h), parasites are unable to extend the IDC and become desynchronized (figure 3, electronic supplementary material, figure S6). Despite resonating with the host period in the Tc21 treatment, parasites were unable to match the phase observed in both Tc24 and WT24 hosts (figure 3*b*), exhibited lower per-cycle productivity (figure 4*b*) and reduced transmission potential (figure 4*e*). However, the lower per-cycle productivity of parasites in Tc21 hosts is compensated for by more cycles occurring during infections, allowing parasites to reach similar overall numbers to those infecting hosts with a 24 h T-cycle (figure 4). By contrast, desynchronization of the IDC rhythm in Tc27 hosts does not impact on per-cycle productivity (figure 4*b*) but there was a trend for reduced transmission potential (figure 4*e*). This suggests that both very high and very low IDC synchrony may be equally fit alternative strategies, or that parasites in long T-cycle hosts prioritize in-host replication over between-host transmission. We also find that the duration of the T-cycle does not affect the severity of infection experienced by canonical circadian clock-disrupted hosts (figure 5).

That the IDC can align to 21 h host rhythms is in keeping with previous observations that *P. chabaudi* shortens the IDC duration by 2–3 h when the temporal alignment between host and parasite rhythms is perturbed, enabling parasites to realign to host rhythms within a few replication cycles [10,40]. While the average period estimate in Tc21 hosts of 21.17 h is shorter than 22 h, it is challenging to accurately estimate the period from short time-series data and resolution is also limited by the sampling interval (four-hourly in this case). Nonetheless, it is clear that the IDC is faster in Tc21 than in Tc24 and WT24 hosts. While our focus was to probe the capacity of parasites to alter the period of the IDC when experiencing within-host environments with different T-cycles, we additionally examined the phase. That the (peak) phase of ring stages is inverted in Tc21 compared to Tc24 and WT24 hosts was unexpected, given the wealth of evidence that the IDC is phased to align with host feeding–fasting rhythms. Our previous studies reveal that the ring stage peaks towards the end of the dark phase in infections initiated in the same type of hosts as the WT24 group [10,19,41,42], yet, in the present study, rings peak at the start of the dark phase in both Tc24 and WT24 hosts. It is unclear why we observed a different phase from previous studies, but one possible clue is that the ‘sampled’ Tc24 and WT24 mice (from which we obtained IDC data) may have similarly reduced rhythmicity to the ‘probed’ Tc24 mice (from which we obtained host rhythms data), impacting on the accuracy with which parasites set the phase of the IDC. Nonetheless, given that the IDCs in both Tc24 and WT24 hosts were rhythmic with *ca* 24 h periods and taking their same phases as a baseline, this suggests the Tc21 parasites have phase-shifted. It would be surprising if different phase-relationships to host rhythms are adaptive for parasites in different T-cycles. Instead, a potential explanation is that having found themselves misaligned to host rhythms upon entering a Tc21 host, parasites are only able to accelerate the IDC by 2 h each cycle, causing the phase of rings to shift relative to host Zeitgeber Time (ZT) over sequential cycles. Under this scenario, if the first 22 h IDC in Tc21 hosts followed that in control (Tc24 and WT24) hosts with the ring stage peak at approximately ZT14, and then gained an additional hour in each cycle, they would reach ZT1−2 by the eighth or ninth IDC (14+8 = 22, which is ZT1), matching the phase estimate from our model fits. This hypothesis can be tested by following the IDC over a longer time series, using the approach in [10], and a within-host model could explore the relationship between plasticity in period and phase and IDC stage durations.

In keeping with previous studies examining how parasites realign to host rhythms following a perturbation to phase, parasites in Tc21 hosts experienced fitness costs. Previous work has revealed that *P. chabaudi* completes an additional replication cycle compared to control infections (in 24 h hosts) during the time it takes to realign following 12 h mismatch to host rhythms [10]. Yet, this additional IDC does not result in more parasites, suggesting that a faster IDC causes parasites to produce fewer progeny per cycle and/or their progeny are less efficient at invading RBC. That Tc21 parasites complete 1–2 more LD cycles than Tc24 and Tc27 parasites during the monitoring window, but their density does not exceed that of control infections (figure 4*a*), suggests that similar costs reduce productivity per LD cycle (figure 4*b*). Constraints on within-host replication are costly for parasite fitness because replication rate is a key determinant of within-host survival, mediating safety in numbers against immune defences and antimalarials, as well as competitive ability in mixed genotype infections [43]. If parasites are only able to shorten the period when facing misalignment to host rhythms, this means they are unable to lengthen the IDC to resonate with Tc27 hosts. This apparent inability is surprising, given that parasite species within the same genus have IDC periods far greater than 24 h (e.g. 48 or 72 h, [8]). However, little is known about the limits to within-species plasticity in period in *Plasmodium*. Future experiments may be able to characterize the IDC rhythm earlier in Tc27 infections before it becomes too damp to estimate the period, revealing whether parasites attempt to resonate with Tc27 host rhythms. A loss of synchrony, as observed in Tc27 infections, is an inevitable consequence if the parasite’s time-keeping mechanism is unable to detect or respond to time cues that occur 27 h apart, and so, parasites free-run. Furthermore, oscillators with a wide range of environmental periods over which they can entrain are expected to be weak, struggling to synchronize [44]. A previous study found that *P. chabaudi* gene expression remained rhythmic in hosts with a 25.7 h period but did dampen (by 62%) after six host cycles [45]. The longer host period plus the longer duration of infections in the present study may have exacerbated this dampening. Slowing the IDC to 27 h may also be suboptimal for the parasite simply because it reduces the replication rate [20]. Additionally, altering period *per se* can affect physiology. For example, grey mouse lemurs exhibit increased energy expenditure and reduced cognitive performance when experiencing a 26 h T-cycle [46].

We also examined the between-host transmission component of parasite fitness (the density of gametocytes in blood correlates positively with the probability of infecting the mosquito vector [47]), finding that parasites in Tc21 hosts exhibit a lower per-cycle gametocyte density (figure 4*e*) mirroring the cost to their within-host replication. The proportion of asexual stages that commit to producing gametocytes is a highly plastic trait [47], so a reduction in gametocyte investment is not necessarily an inevitable consequence of fewer asexually replicating stages. *Plasmodium* parasites reduce their commitment to gametocytes when experiencing stressful conditions in the host as a form of reproductive restraint, and they maximize commitment when exposed to catastrophic conditions as a form of terminal investment [48,49]. Thus, it is possible that parasites in Tc21 hosts adopt reproductive restraint to maintain in-host replication in response to the (non-catastrophic) costs of a short IDC duration and/or being out of phase with host rhythms [50]. More data are required to probe whether the non-significant trend for fewer gametocytes in Tc27 infections (figure 4*d*,*e*) also represents adaptive reproductive restraint. Furthermore, gametocytes produced from mis-phased asexual stages are likely to be less infective to mosquitoes [13,14], exacerbating the reduction in transmission potential experienced by Tc21 and Tc27 parasites.

That the severity of infections did not vary across T-cycle treatments (in *Per1/2*-null hosts) was unexpected given the myriad negative consequences of circadian disruption for health, and of phenological mismatch between organisms in the wild. A likely explanation is that the lack of canonical circadian clock in *Per1/2*-null mutants protected them from the adverse effects of attempting to establish and maintain alignment of rhythms across multiple cell, tissue and organ types in non-24 h T-cycles [51–53]. Furthermore, while most feeding, across all T-cycles, was nocturnal, unrestricted access to food may have enabled hosts to maximize their physiological condition, ameliorating any T-cycle-specific costs of infection. While our experiment was not designed to differentiate between any potentially independent impacts of LD cycle number and the absolute duration of infection (HPI) on severity, our analyses suggest that symptoms correlate best with the duration of infection. If supported by future work, this suggests that the cumulative length of time for which a host is infected is more important than rhythmic impacts on physiology for severity. Under this scenario, interventions that interfere with parasite rhythms and have knock-on effects on host rhythms are unlikely to negatively affect host health. Such interventions could coerce parasites into shortening the IDC to reduce their replication and transmission. Even a small reduction in parasite density could allow drugs and immune response to be more effective. Given that out-of-the-box solutions to malaria are urgently needed, the IDC rhythm is a potential target. For such interventions to be robust against parasite counter-evolution, understanding the costs, benefits and limits on plasticity in the IDC is necessary [54].

## Data Availability

The datasets supporting the findings of this study are deposited in the University of Edinburgh’s DataShare repository [55]. Supplementary material is available online [56].
